# Predicting sepsis onset in ICU using machine learning models: a systematic review and meta-analysis

**DOI:** 10.1186/s12879-023-08614-0

**Published:** 2023-09-27

**Authors:** Zhenyu Yang, Xiaoju Cui, Zhe Song

**Affiliations:** 1 Kunming Medical University, Kunming, Yunnan China; 2 Chengyang District People’s Hospital, Qingdao, Shandong China; 3 Qinghai University, Xining, Qinghai China

**Keywords:** Machine learning, Sepsis, Intensive care units, Meta-analysis

## Abstract

**Background:**

Sepsis is a life-threatening condition caused by an abnormal response of the body to infection and imposes a significant health and economic burden worldwide due to its high mortality rate. Early recognition of sepsis is crucial for effective treatment. This study aimed to systematically evaluate the performance of various machine learning models in predicting the onset of sepsis.

**Methods:**

We conducted a comprehensive search of the Cochrane Library, PubMed, Embase, and Web of Science databases, covering studies from database inception to November 14, 2022. We used the PROBAST tool to assess the risk of bias. We calculated the predictive performance for sepsis onset using the C-index and accuracy. We followed the PRISMA guidelines for this study.

**Results:**

We included 23 eligible studies with a total of 4,314,145 patients and 26 different machine learning models. The most frequently used models in the studies were random forest (*n* = 9), extreme gradient boost (*n* = 7), and logistic regression (*n* = 6) models. The random forest (test set *n* = 9, acc = 0.911) and extreme gradient boost (test set *n* = 7, acc = 0.957) models were the most accurate based on our analysis of the predictive performance. In terms of the C-index outcome, the random forest (*n* = 6, acc = 0.79) and extreme gradient boost (*n* = 7, acc = 0.83) models showed the highest performance.

**Conclusion:**

Machine learning has proven to be an effective tool for predicting sepsis at an early stage. However, to obtain more accurate results, additional machine learning methods are needed. In our research, we discovered that the XGBoost and random forest models exhibited the best predictive performance and were most frequently utilized for predicting the onset of sepsis.

**Trial registration:**

CRD42022384015

**Supplementary Information:**

The online version contains supplementary material available at 10.1186/s12879-023-08614-0.

## Introduction

Sepsis is a severe and potentially life-threatening condition resulting from a dysregulated immune response to infection [1]. Early detection and prompt treatment are crucial for improving patient outcomes and reducing health care costs. In recent years, machine learning (ML) models have emerged as promising tools for detecting and managing sepsis in the intensive care unit (ICU) [2]. These models use complex algorithms and statistical methods to learn from large volumes of patient data, including vital signs, laboratory results, and electronic health records, and to predict the onset of sepsis before its clinical manifestations become apparent [3]. The early identification and treatment of sepsis are related to the improvement of patient prognosis. Machine learning-based warning systems may shorten recognition time. Adams R et al. [4] set up a system called the “Targeted Real-time Early Warning System”, and they found that early warning systems have the potential to identify sepsis patients early and improve their prognosis and can identify and prioritize sepsis patients who would benefit the most from early treatment. By enabling early detection, ML models hold tremendous potential for enhancing patient care and reducing the burden of sepsis on health care systems worldwide.

The Sepsis-3 definitions suggest that patients with at least two of the following three clinical variables may be prone to the poor outcomes typical of sepsis: (1) a low blood pressure (SBP ≤ 100 mmHg), (2) a high respiratory rate (≥ 22 breaths per min), or (3) altered mentation (Glasgow coma scale score < 15). Machine learning can utilize computers to review a large number of clinical cases, and mature machine learning models can be used to make real-time evaluations of whether patients will develop sepsis, allowing for immediate intervention.

In this study, we aimed to explore the use of ML models for predicting the onset of sepsis in the ICU. Specifically, we reviewed the literature on ML models for sepsis prediction, highlighting their strengths and limitations. Additionally, in this article, we discuss the potential impact of these models on patient outcomes, clinical decision-making, and health care costs. Through this meta-analysis, we hope to shed light on the promise of ML models as tools for improving the management of sepsis in the ICU and beyond.

## Methods

### Study design and literature search

This study retrieved relevant studies on the timing of sepsis diagnosis by machine learning  from the Cochrane Library, Embase, PubMed, and Web of Science databases and extracted data from these studies. The Cochrane Library, Embase, PubMed and Web of Science databases were searched from inception to 14/11/2022. Search formulas were constructed based on combinations of MeSH headings and free words. We did not put any restriction on the language or region. The literature search was completed by Zhenyu Yang and Xiaoju Cui (the search detail is shown in Supplementary file 2). All selected studies were imported to EndNote 2020. We filtered studies according to the abstract. Duplicate articles were deleted. Literature screening was independently performed by two reviewers (Zhenyu Yang and Xiaoju Cui). Any disagreement was settled by a third reviewer. The retrieval formular file is presented in Supplementary material 2.

### Inclusion and exclusion criteria

Inclusion criteria.

1. Randomized controlled trials (RCTs), prospective cohort studies, and nested case‒control studies.
2. Studies in which the predictive model was completely established.

Exclusion criteria.

1. Studies unrelated to sepsis
2. Studies with incomplete data
3. Studies in which the outcome measures related to the effectiveness of predictive measures were not included.
4. Animal studies, reviews, conference abstracts, guidelines, letters, comments, and meta-analyses
5. Non-RCT research designs
6. Non-English articles
7. Basic articles on pathology, physiology, and biochemistry
8. Duplicate publications

### Data extraction

The data extraction form was detailed according to the Modified CHARMS checklist. The checklist included the name of the first author, publication date, nationality, duration of data collection, study design, type of validation (internal, external, random split and time split) and sample size (total number of participants, development and testing clusters).

### Risk of bias assessment

We used PROBAST and an external prognostic validity model to assess the risk of bias of the selected studies [5]. PROBAST is a checklist designed for systemic reviews of diagnostic or prognostic prediction models. The risk of bias was assessed independently by two reviewers (Zhe Song and Zhenyu Yang). PROBAST consists of two parts: A. an overall bias risk assessment (including research objects, predictors, results and statistical methods) and B. an overall applicability assessment (research objects, predictors and results).

### Statistical analysis

We performed descriptive statistics to summarize the characteristics of the models. For prediction models that were evaluated in more than two independent datasets, a random effect meta-analysis was conducted to estimate their performance and accuracy. If a measure of uncertainty, such as the standard error or 95% confidence interval, was unavailable for the mean C-index, we computed it based on the number of events and participants. All data analyses were carried out using R software version 4.1.1.

## Results

### Study selection

**Fig. 1:**
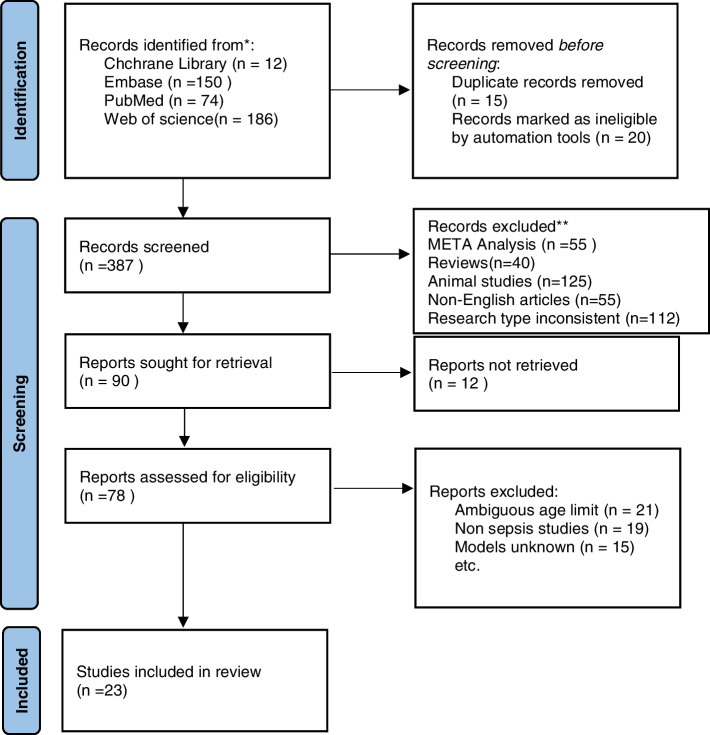
PRISMA Study Selection Flowing Chart. This figure is a flowchart of the inclusion article after screening based on the inclusion and exclusion criteria in this study

A total of 422 articles were identified through various databases, including the Cochrane Library (*n* = 12), Embase (*n* = 150), PubMed (*n* = 74), and Web of Science (*n* = 186) databases. After eliminating 15 duplicate articles and excluding ineligible records using automation tools, we browsed 387 articles. Ultimately, 23 articles met the inclusion criteria and were included in our study [2, 6–27]. Figure 1 displays the PRISMA flow diagram illustrating our study selection process. The selection was conducted independently by two reviewers (Zhenyu Yang and Xiaoju Cui). Any discrepancies were resolved by a third reviewer.

### Characteristics of included studies

A total of 1,287,160 individuals were included in this study, with 167,338 individuals included in the validation set. All articles analysed were published within the past 5 years, indicating a growing interest in the use of machine learning for sepsis prediction. Our research identified 81 prognostic models, including 5 based on deep learning, 4 based on InSight, 10 based on logistic regression, 6 based on multilayer perceptron, 8 based on neural networks, 8 based on support vector machines, 14 based on XGBoost, 15 based on random forest, and 11 based on SOFA. Detailed characteristics of the included studies can be found in Table 1.

**Table 1:** Detailed characteristics of the included studies

**Studies**	**First Author**	**Nation**	**Study Type**	**Sample source**	**Disease background**	**Diagnosis of sepsis**	**Missing data**	**Model type**
SSP: Early prediction of sepsis using fully connected LSTM-CNN model	Alireza Rafiei2020	Iran	Retrospective Cohort	Retrospectively	≥ 14y patients and ICU LOS > 10d	Sepsis 3.0	Multiple imputation	SSP-LSTM, SSP-GRU, InSight, AISE
Evaluation of a machine learning algorithm for up to 48-h advance prediction of sepsis using six vital signs	Christopher Barton2019	U.S.A	Prospectively	Prospectively	≥ 18y patients	Sepsis 3.0	Multiple imputation	MLA、SIRS、MEWS、SOFA
Predicting 30-days mortality for MIMIC-III patients with sepsis-3: a machine learning approach using Xgboost	Nianzong Hou2020	P.R.C	Prospectively	Prospectively	≥ 18y patients and ICU LOS > 24d	Sepsis 3.0	Delete	XGBoost, LR,SAPS-II
A machine learning-based model for 1-year mortality prediction in patients admitted to an Intensive Care Unit with a diagnosis of sepsis	J E García-Gallo2018	Colombia	Retrospectively	Retrospectively	≥ 16y patients	Sepsis 3.0	Multiple imputation	SGB、OASIS、SOFA、SAPS2
Interpretable Machine Learning for Early Prediction of Prognosis in Sepsis: A Discovery and Validation Study	Chang Hu2022	P.R.C	Prospectively	Prospectively	≥ 18y patients and ICU LOS > 24d	Sepsis 3.0	Multiple imputation	SVM、KNN、XGBoost、DT、RF、NB、LR
Machine Learning Model to Identify Sepsis Patients in the Emergency Department: Algorithm Development and Validation	Lin PC2021	P.R.C	Retrospective Cohort	Retrospective Cohort	≥ 20y patients	Sepsis 3.0	Multiple imputation	XGBoost、SIRS、SOFA
Dynamic Sepsis Prediction for Intensive Care Unit Patients Using XGBoost-Based Model With Novel Time-Dependent Features	Shuhui Liu2017	P.R.C	Retrospective Cohort	Retrospective Cohort	≥ 18y patients	Sepsis 3.0	Multiple imputation	RF、GRU、CNNLSTM、EASP
Effect of a machine learning-based severe sepsis prediction algorithm on patient survival and hospital length of stay: a randomised clinical trial	Shimabukuro DW2018	U.S.A	Retrospective Cohort	Retrospective Cohort	≥ 18y patients	Sepsis 3.0	Multiple imputation	MLA、SIRS、MEWS、SOFA
A Predictive Model Based on Machine Learning for the Early Detection of Late-Onset Neonatal Sepsis: Development and Observational Study	Wongeun Song2022	KOREA	Retrospective Cohort	Retrospective Cohort	≥ 18y patients	Sepsis 3.0	Multiple imputation	RF、LR、SVM、NB、XGBOOST
Machine learning approach for the prediction of 30-day mortality in patients with sepsis-associated encephalopathy	Liwei Peng2022	P.R.C	Retrospective Cohort	Retrospective Cohort	≥ 18y patients	Sepsis 3.0	Multiple imputation	CS、MIG、LLI、ET、RF、GB
Development and validation of a novel blending machine learning model for hospital mortality prediction in ICU patients with Sepsis	Zhixuan Zeng2021	P.R.C	Retrospective Cohort	Retrospective Cohort	≥ 18y patients	Sepsis 3.0	Multiple imputation	SAPS II、SOFA、LR、LDA、CART、NB、KNN、MLP、SVM、RF、XGB
Machine learning predicts mortality in septic patients using only routinely available ABG variables: a multi-centre evaluation	Bernhard Wernly2021	Austria	Retrospective Cohort	Retrospective Cohort	≥ 18y patients	Sepsis 3.0	Multiple imputation	LR、LSTM、SOFA
A Machine Learning Model for Accurate Prediction of Sepsis in ICU Patients	Dong Wang2021	P.R.C	Retrospective Cohort	Retrospective Cohort	≥ 18y patients	Sepsis 3.0	Multiple imputation	RF
Early Prediction of Mortality, Severity, and Length of Stay in the Intensive Care Unit of Sepsis Patients Based on Sepsis 3.0 by Machine Learning Models	Longxiang Su2021	Germany	Retrospective Cohort	Retrospective Cohort	≥ 18y patients	Sepsis 3.0	Multiple imputation	LR、RF、XGBoost
Supervised classification techniques for prediction of mortality in adult patients with sepsis	Rodríguez A2021	Colombia	Retrospective Cohort	Retrospective Cohort	≥ 18y patients	Sepsis 3.0	Multiple imputation	DT、RF、NN、SVM
A Machine Learning Sepsis Prediction Algorithm for Intended Intensive Care Unit Use (NAVOY Sepsis): Proof-of-Concept Study	Inger Persson2021	Sweden	Retrospective Cohort	Retrospective Cohort	≥ 18y patients	Sepsis 3.0	Multiple imputation	NAVOY
Development of a Nomogram to Predict 28-Day Mortality of Patients With Sepsis-Induced Coagulopathy: An Analysis of the MIMIC-III Database	Zongqing Lu2021	P.R.C	Retrospective Cohort	Retrospective Cohort	≥ 18y patients	Sepsis 3.0	Multiple imputation	Nomogram 、SOFA、LODS、SAPS II、SIC score
A Simple Weaning Model Based on Interpretable Machine Learning Algorithm for Patients With Sepsis: A Research of MIMIC-IV and eICU Databases	Wanjun Liu2022	P.R.C	Retrospective Cohort	Retrospective Cohort	≥ 18y patients	Sepsis 3.0	Multiple imputation	XGBOOST 、MLP、RF 、SVM 、LR 、KNN
The development an artificial intelligence algorithm for early sepsis diagnosis in the intensive care unit	Yuan KC2020	P.R.C	Retrospective Cohort	Retrospective Cohort	≥ 18y patients	Sepsis 3.0	Multiple imputation	XGBoost、SOFA
Early diagnosis of bloodstream infections in the intensive care unit using machine-learning algorithms	Michael Roimi2019	Israel	Retrospective Cohort	Retrospective Cohort	≥ 18y patients	Sepsis 3.0	Multiple imputation	RF
Predicting sepsis with a recurrent neural network using the MIMIC III database	Matthieu Scherpf2019	Germany	Retrospective Cohort	Retrospective Cohort	≥ 18y patients	Sepsis 3.0	Multiple imputation	RNN、InSight
Predicting central line-associated bloodstream infections and mortality using supervised machine learning	Joshua P. Parreco20182018	U.S.A	Retrospective Cohort	Retrospective Cohort	≥ 19y patients	Sepsis 3.1	Multiple imputation	LR、GBT、DL
Multicentre validation of a sepsis prediction algorithm using only vital sign data in the emergency department, general ward and ICU	Qingqing Mao2018	U.S.A	Retrospective Cohort	Retrospective Cohort	≥ 18y patients	Sepsis 3.0	Multiple imputation	InSight、 MEWS 、SOFA、 SIRS

**Studies**	**Train set sepsis number**	**Train set number**	**Testing set**	**Method of testing**	**Test set sepsis number**	**Test set number**	**Number of variables**	**Outcome indicators**
SSP: Early prediction of sepsis using fully connected LSTM-CNN model	2542	20336	1	Multicenter	2500	20000	14	AUROC, Sensitivity Specificity
Evaluation of a machine learning algorithm for up to 48-h advance prediction of sepsis using six vital signs	2649	91445	1	Multicenter	1024	21507	4	AUROC Sensitivity Specificity
Predicting 30-days mortality for MIMIC-III patients with sepsis-3: a machine learning approach using Xgboost	10704	46520	1	Multicenter	889	4559	12	AUROC
A machine learning-based model for 1-year mortality prediction in patients admitted to an Intensive Care Unit with a diagnosis of sepsis	46520	58977	1	Multicenter	5650	15254	18	AUROC
Interpretable Machine Learning for Early Prediction of Prognosis in Sepsis: A Discovery and Validation Study	12292	76540	1	Multicenter	8817	12292	15	AUROC Sensitivity Specificity
Machine Learning Model to Identify Sepsis Patients in the Emergency Department: Algorithm Development and Validation	6637	8296	1	Random sampling	506	1744	26	AUROC Sensitivity Specificity
Dynamic Sepsis Prediction for Intensive Care Unit Patients Using XGBoost-Based Model With Novel Time-Dependent Features	3526	34472	1	Random sampling	4526	34472	30	AUROC Sensitivity Specificity
Effect of a machine learning-based severe sepsis prediction algorithm on patient survival and hospital length of stay: a randomised clinical trial	67	142	0	Single center	——	——	3	AUROC Sensitivity Specificity
A Predictive Model Based on Machine Learning for the Early Detection of Late-Onset Neonatal Sepsis: Development and Observational Study	1572	40366	1	Multicenter	315	1257	21	AUROC Sensitivity Specificity
Machine learning approach for the prediction of 30-day mortality in patients with sepsis-associated encephalopathy	4897	382278	1	Multicenter	2097	382278	15	AUROC Sensitivity Specificity
Development and validation of a novel blending machine learning model for hospital mortality prediction in ICU patients with Sepsis	12558	200859	1	Multicenter	12095	61532	6	AUROC
Machine learning predicts mortality in septic patients using only routinely available ABG variables: a multi-centre evaluation	8061	61532	1	Multicenter	3853	200859	23	Sensitivity Specificity
A Machine Learning Model for Accurate Prediction of Sepsis in ICU Patients	3539	17005	1	Multicenter	910	17005	55	AUROC, Sensitivity Specificity
Early Prediction of Mortality, Severity, and Length of Stay in the Intensive Care Unit of Sepsis Patients Based on Sepsis 3.0 by Machine Learning Models	2436	11512	0	Single center	——	——	26	AUROC, Sensitivity Specificity
Supervised classification techniques for prediction of mortality in adult patients with sepsis	2510	5022	1	Multicenter	2510	5022	27	AUROC, Sensitivity Specificity
A Machine Learning Sepsis Prediction Algorithm for Intended Intensive Care Unit Use (NAVOY Sepsis): Proof-of-Concept Study	2893	61532	0	Single center	——	——	6	AUROC Sensitivity Specificity
Development of a Nomogram to Predict 28-Day Mortality of Patients With Sepsis-Induced Coagulopathy: An Analysis of the MIMIC-III Database	3280	9432	1	Multicenter	987	3280	17	AUROC
A Simple Weaning Model Based on Interpretable Machine Learning Algorithm for Patients With Sepsis: A Research of MIMIC-IV and eICU Databases	5020	10832	1	Multicenter	7081	33790	20	AUROC Sensitivity Specificity
The development an artificial intelligence algorithm for early sepsis diagnosis in the intensive care unit	319	1588	0	Single center	——	——	19	AUROC Sensitivity Specificity
Early diagnosis of bloodstream infections in the intensive care unit using machine-learning algorithms	1021	1812	1	Multicenter	2351	7419	29	AUROC Sensitivity Specificity
Predicting sepsis with a recurrent neural network using the MIMIC III database	4278	58976	0	Single center	——	——	10	AUROC
Predicting central line-associated bloodstream infections and mortality using supervised machine learning	22201	57786	0	Single center	——	——	37	AUROC Sensitivity Specificity
Multicentre validation of a sepsis prediction algorithm using only vital sign data in the emergency department, general ward and ICU	1179	21604	0	Single center	——	——	7	AUROC Sensitivity Specificity

This table provides detailed information on the various studies included in this study

### Quality assessment

**Fig. 2:**
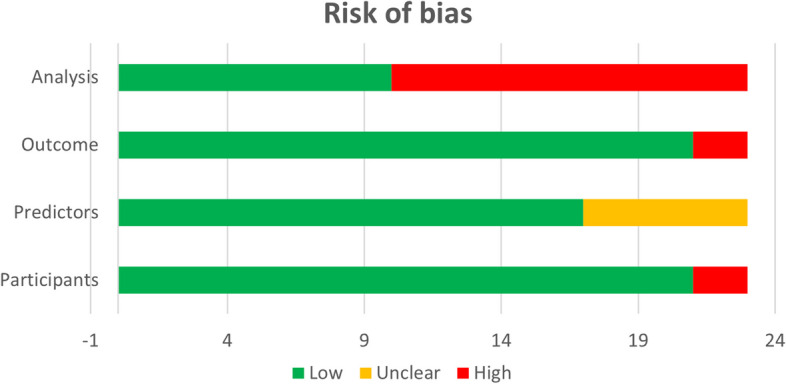
Risk of Bias Assessment. This figure illustrates the risk bias included in this study

The quality assessment was conducted independently by two reviewers (Zhenyu Yang and Xiaoju Cui), and any discrepancies were resolved by a third reviewer. The results of the quality assessment are presented in the risk of bias picture (Fig. 2). Two studies (8.6%) were deemed to have a high risk of bias in the participant domain, 13 studies (58.3%) were deemed to have a high risk of bias in the analysis domain, and two studies (8.6%) were deemed to have a high risk of bias in the outcome domain. No studies were deemed to have a high risk of bias in the predictor domain. A high risk of bias in the analysis domain may be attributed to an inadequate sample size, insufficient events per variable (EPV), improper handling of missing data, or failure to report how missing data were handled. The PRISMA checklist can be found in Supplementary file 1.

### Predictors

**Fig. 3:**
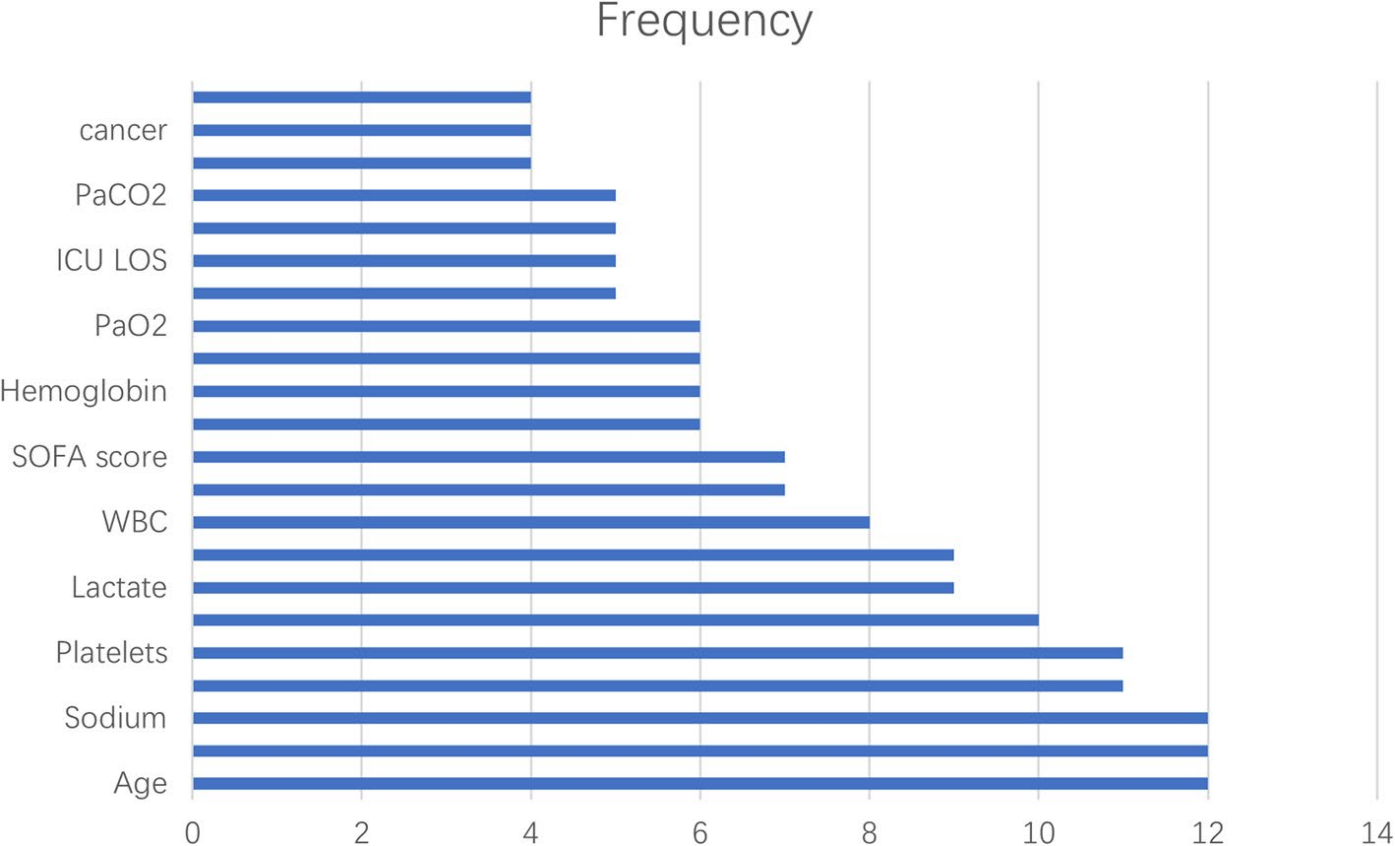
Predictors Frequency Bar Chart. This figure indicates the number of times the items on the left side of the figure were used as indicators in the included literature

Age, creatinine levels, and sodium levels were the most frequently used predictors (*n* = 12), followed by blood pressure and platelet levels (*n* = 11) and heart rate (*n* = 9). The remaining predictors were ranked in descending order of frequency as follows: lactate levels and temperature (*n* = 9), the WBC count (*n* = 8), the respiratory rate and SOFA score (*n* = 7), glucose, haemoglobin, MCHC, and PaO2 levels (*n* = 6), the GCS score, ICU LOS, lymphocyte count, and PaCO2 levels (*n* = 5), and BUN levels, cancer, and sex (*n* = 4). These results are presented in Fig. 3.

### Training set and test set accuracy

**Fig. 4:**
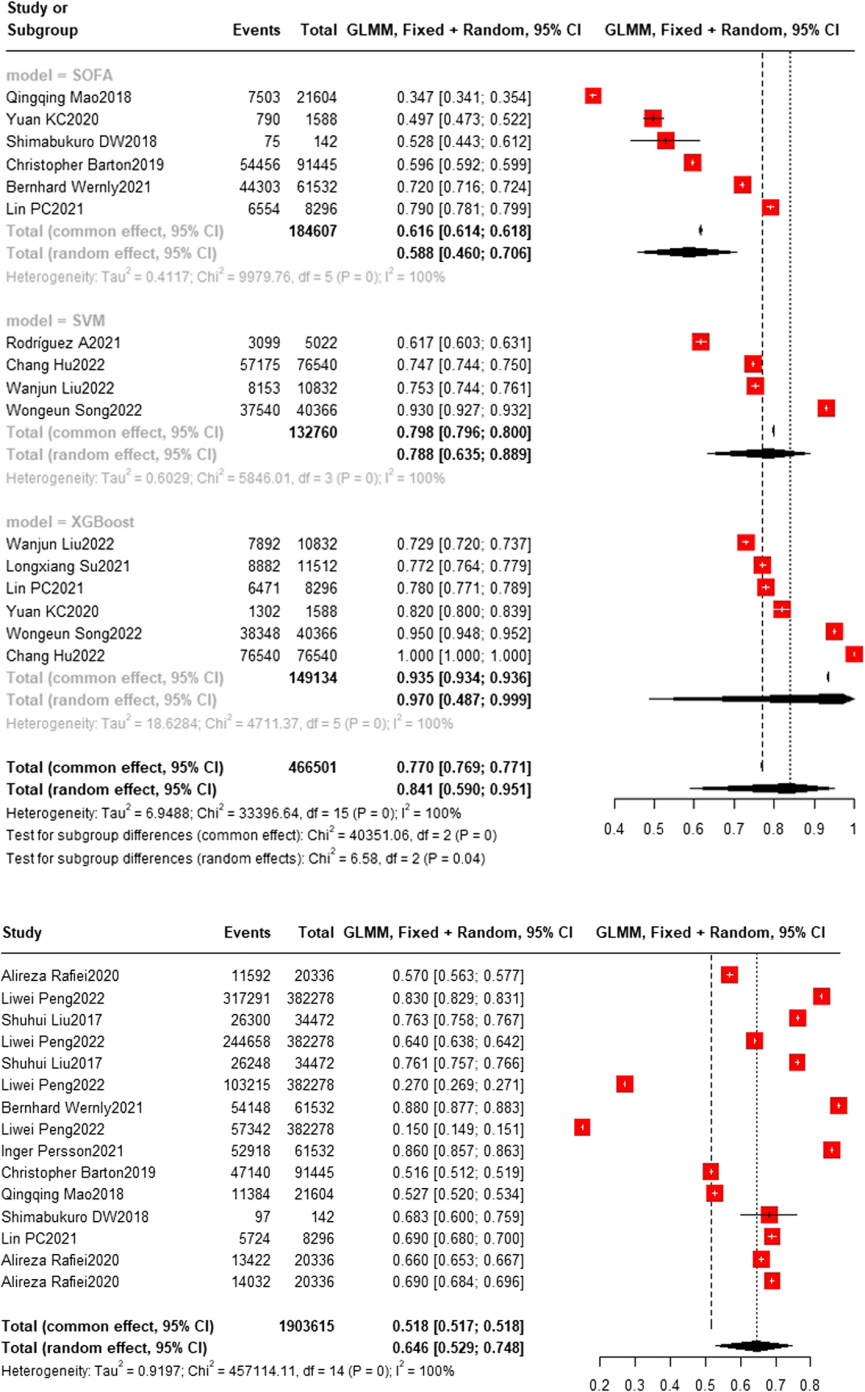
Train set accuracy. In the train set, XGBoost showed the best predicting performance (*n* = 6), with an accuracy of 0.970 (0.487, 0.999) The accuracy of SOFA model (*n* = 6) is 0.588 (0.460,0.706). The accuracy of SVM model (*n* = 4) is 0.788 (0.635,0.889) The accuracy of XGBoost model (*n* = 6) is 0.970 (0.487,0.999)

**Fig. 5:**
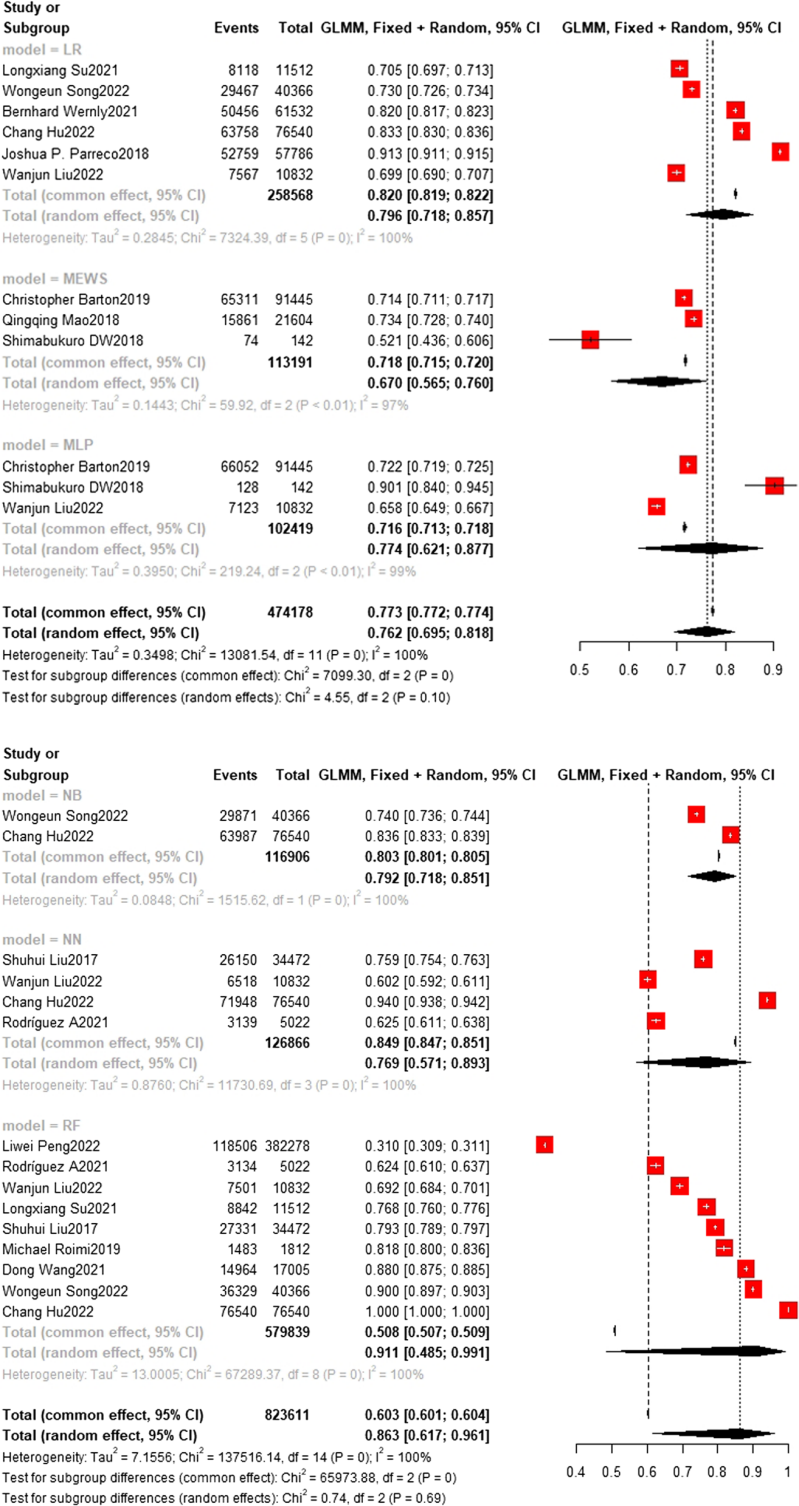
Train set accuracy. In the train set, the Random Forest model was the most frequently applied machine learning model (*n* = 9), with an accuracy of 0.911 (0.485, 0.991). The accuracy of LR model (*n* = 6) is 0.796 (0.718,0.857) The accuracy of MEWS model (*n* = 3) is 0.670 (0.565,0.760) The accuracy of MLP model (*n* = 3) is 0.774 (0.695,0.818). The accuracy of NB model (*n* = 2) is 0.792 (0.718,0.851) The accuracy of NN model (*n* = 4) is 0.769 (0.571,0.893)

**Fig. 6:**
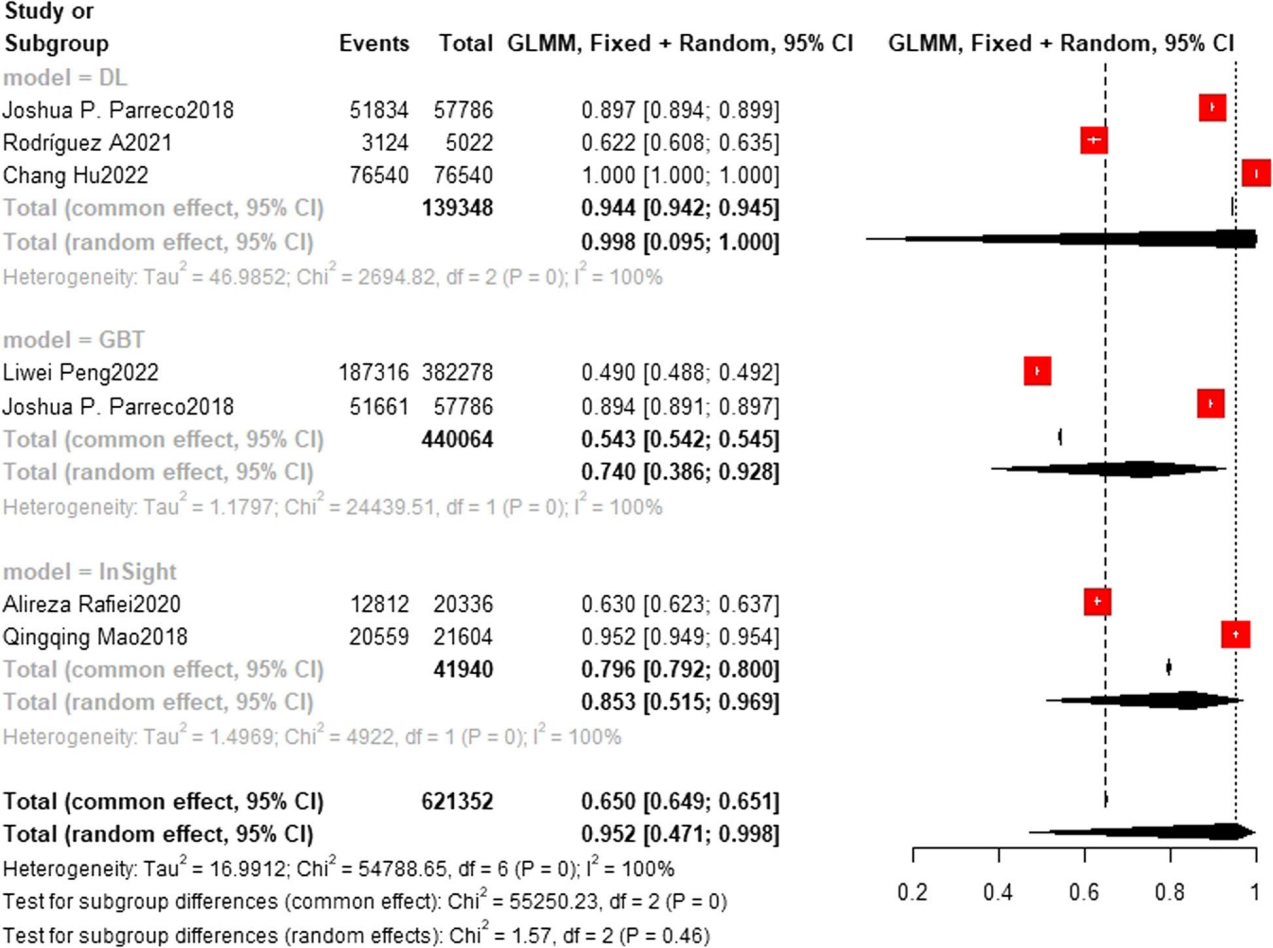
Train set accuracy. The accuracy of DL model (*n* = 3) is 0.998 (0.095,1.000) GBT (*n* = 2) and InSight model (*n* = 2) are 0.740(0.386,0.928) and 0.853(0.515,0.969) respectively

**Fig. 7:**
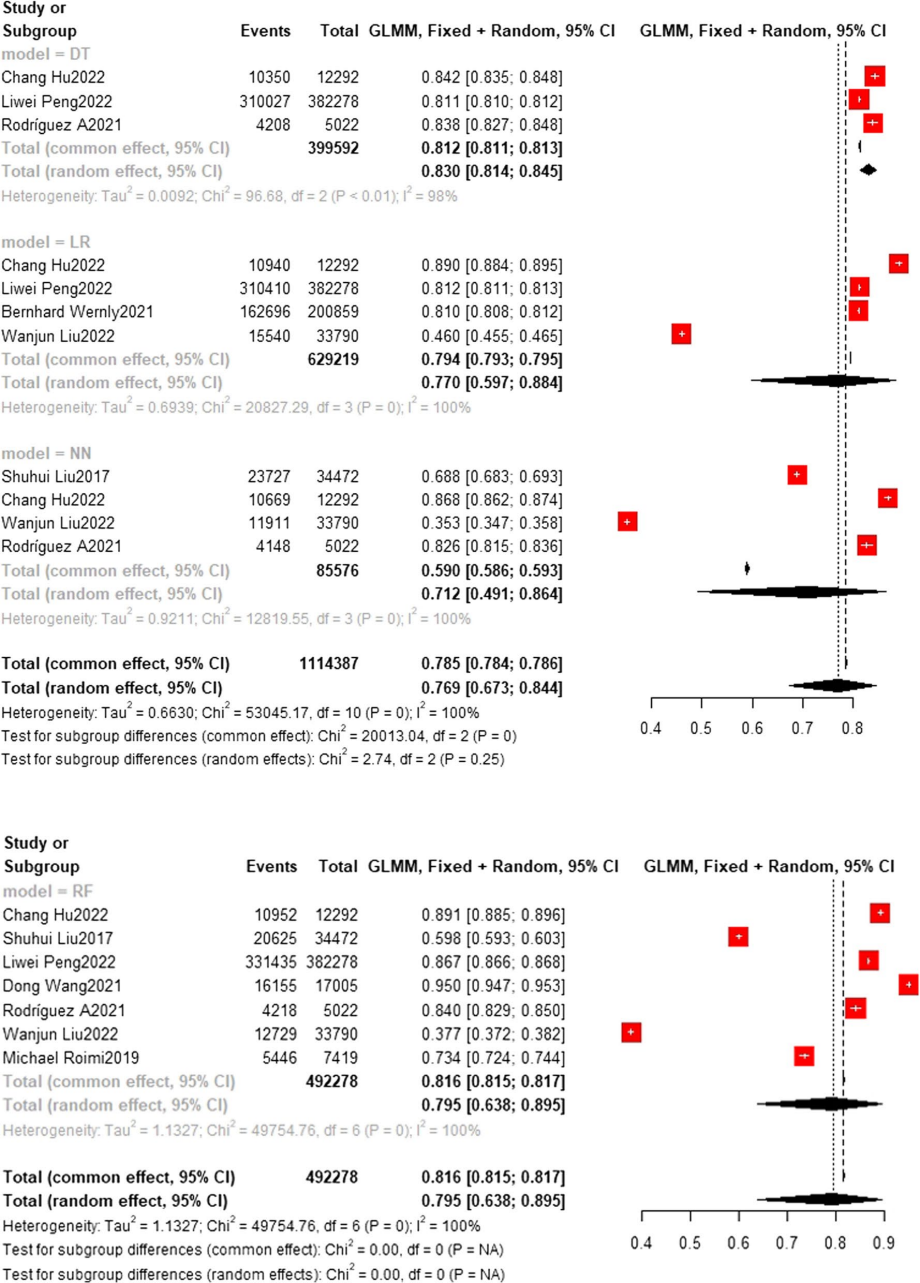
Test set accuracy. In the test set, the Random Forest model was also the most frequently applied machine learning model (n = 7), with an accuracy of 0.795 (0.638, 0.895). The DT model showed the best predicting performance (*n* = 3), with an accuracy of 0.830 (0.814, 0.845). The accuracy of LR model (*n* = 4) and NN model (*n* = 4) are 0.770 (0.597,0.884) and 0.712 (0.491,0.864) respectively

**Fig. 8:**
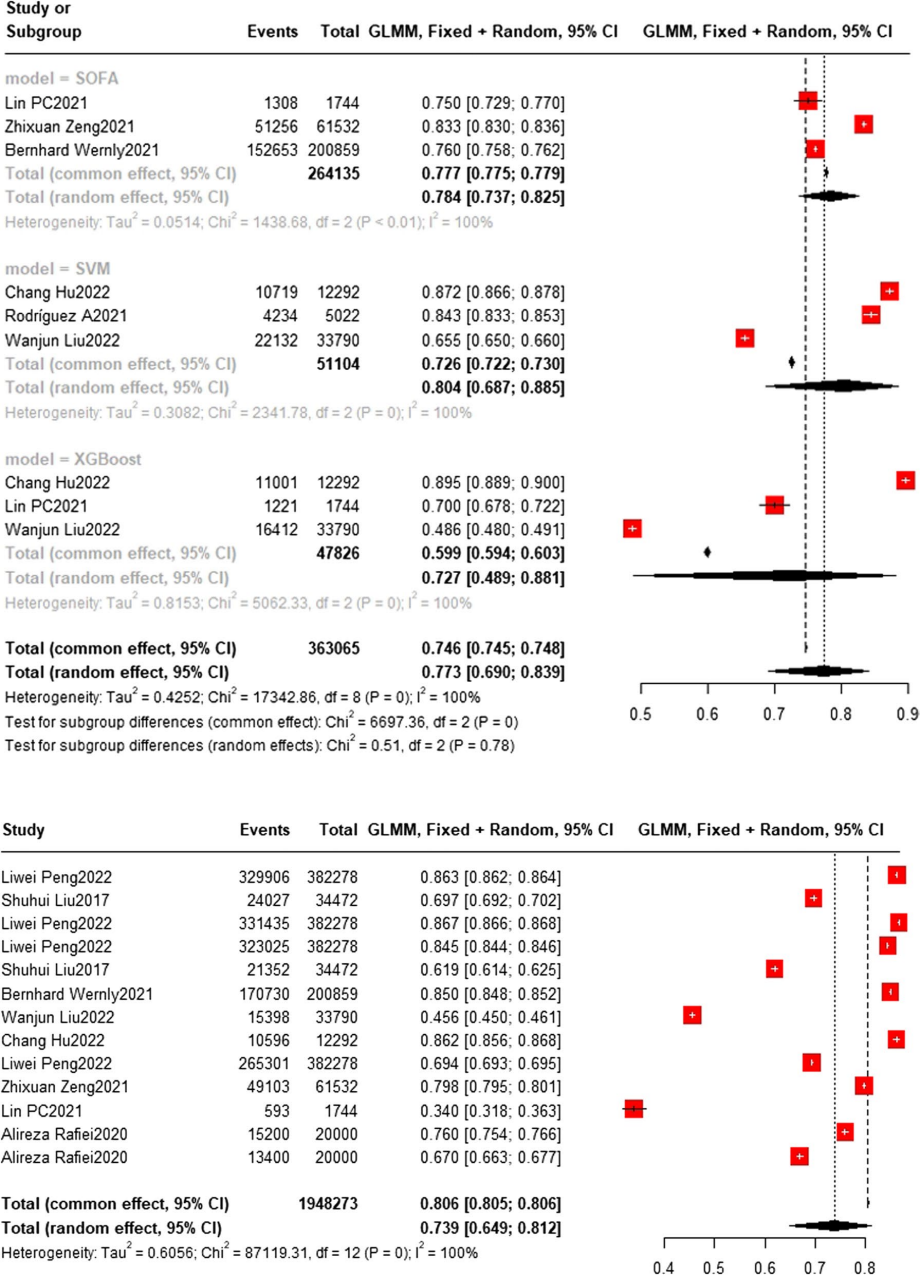
Test set accuracy. The accuracy of SOFA model (*n* = 3) is 0.784 (0.737,0.825) The accuracy of SVM model (*n* = 3) is 0.804 (0.687,0.885). The accuracy of XGBoost model (*n* = 3) is 0.727 (0.489,0.881)

In the training set, the random forest model was the most frequently applied machine learning model (*n* = 9), with an accuracy of 0.911 (0.485, 0.991). The XGBoost model showed the best predictive performance (*n* = 6), with an accuracy of 0.970 (0.487, 0.997). In the test set, the random forest model was also the most frequently applied machine learning model (*n* = 7), with an accuracy of 0.795 (0.638, 0.895). The deep learning model showed the best predictive performance (*n* = 3), with an accuracy of 0.830 (0.814, 0.845). These results are presented in Figs. 4, 5, 6, 7 and 8.

### Training set and test set c-index

**Fig. 9:**
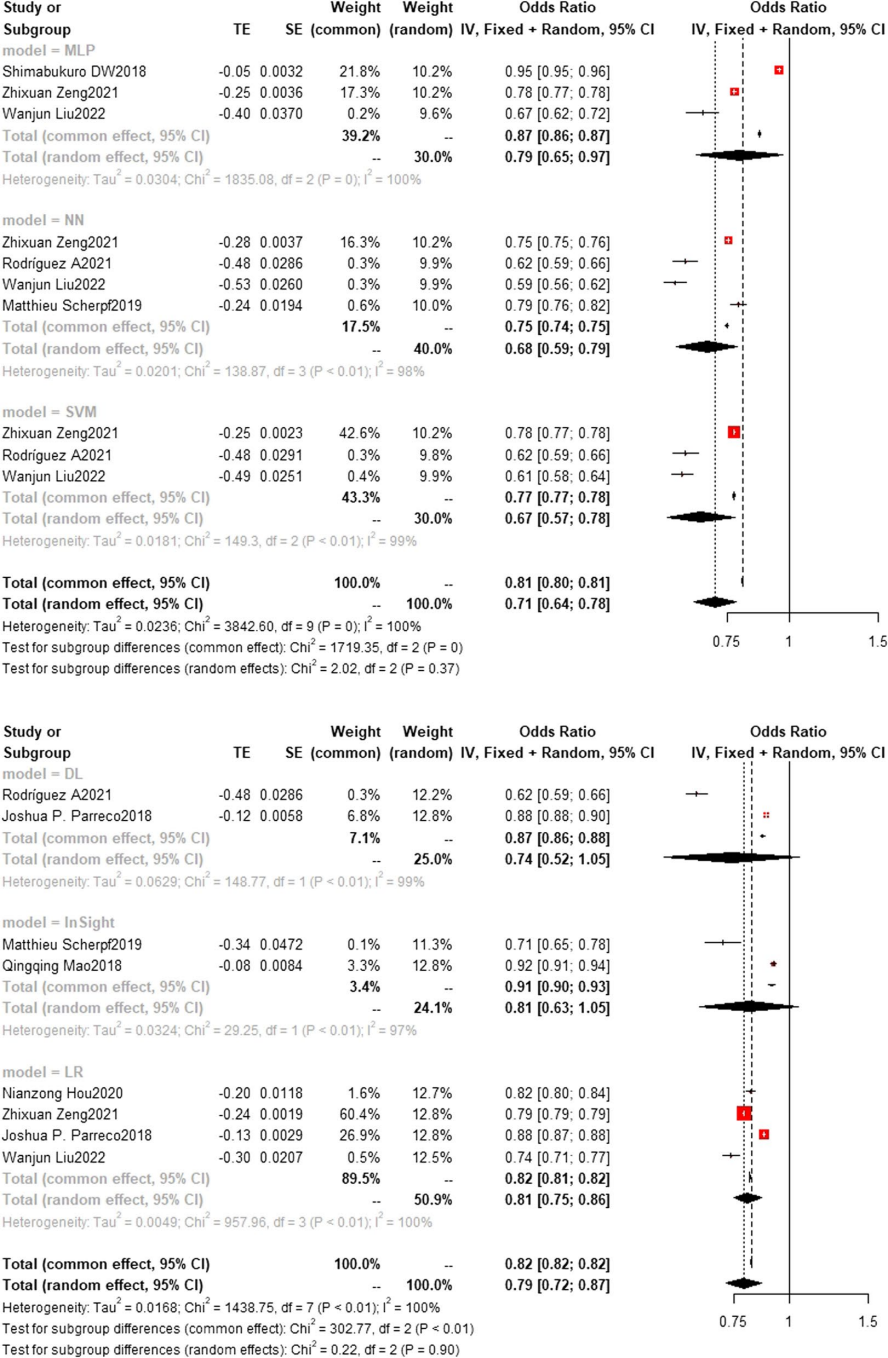
Train set c-index. In the train set, InSight exhibited the best performance with a c-index of 0.91 (0.90,0.93) in 2 studies. The rest are MLP(*N* = 3), NN(*n* = 4), SVM(*n* = 3), DL(*n* = 2) and LR(*n* = 4). the C-index of them are 0.79 (0.65,0.97), 0.68(0.59,0.79), 0.67(0.57,0.78), 0.74(0.52,1.05) and 0.81(0.75,0.86)

**Fig. 10:**
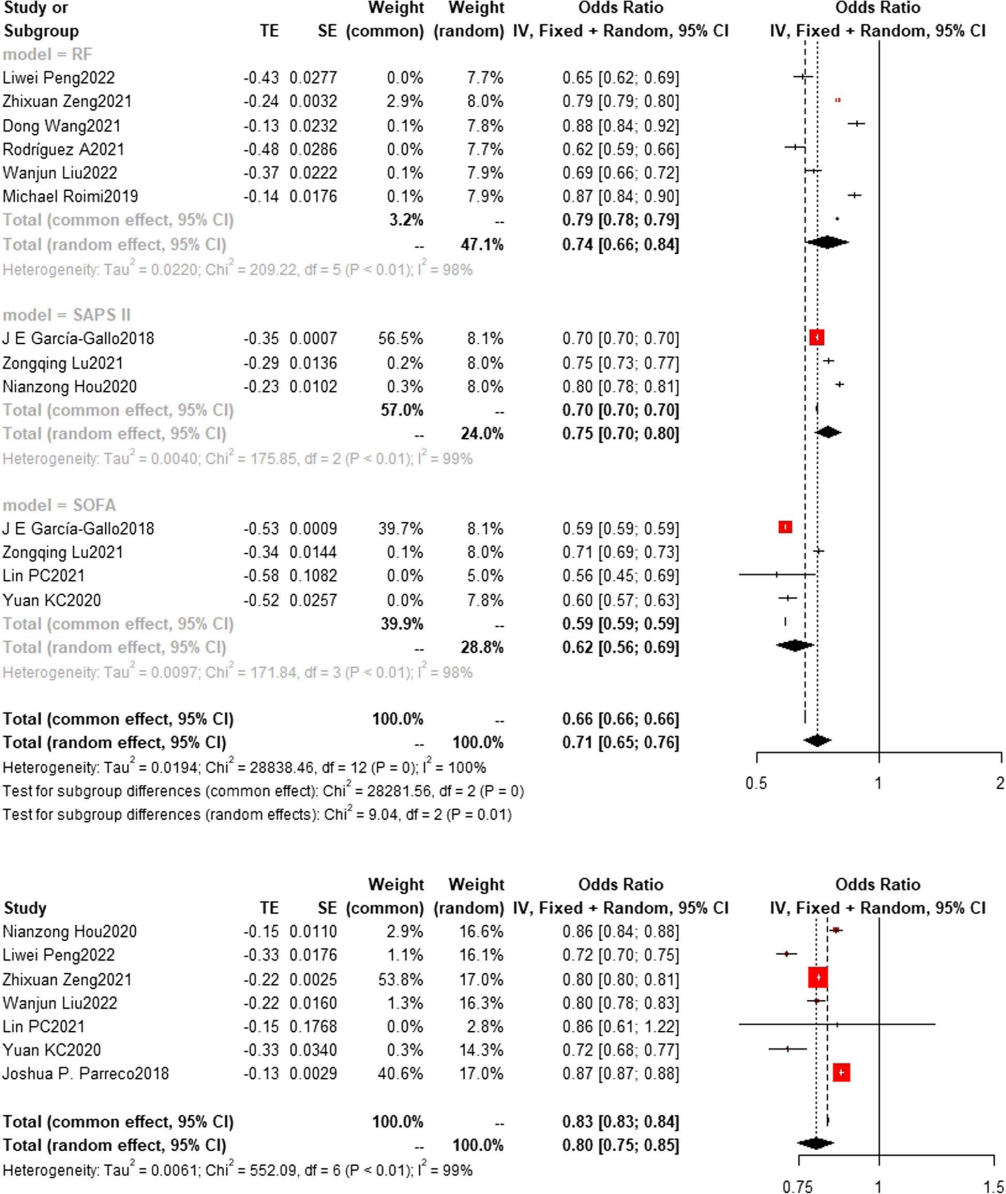
Train set c-index. In the train set, XGBoost (bottom) was the most frequently utilized machine learning model with a c-index of 0.83 (0.83,0.84) in 7 studies. The rest are RF(*n* = 6) SAPS II(*n* = 3) and SOFA(*n* = 4), the C-index of them are 0.79 (0.78,0.79) 0.70(0.70,0.70) and 0.66(0.66,0.66)

**Fig. 11:**
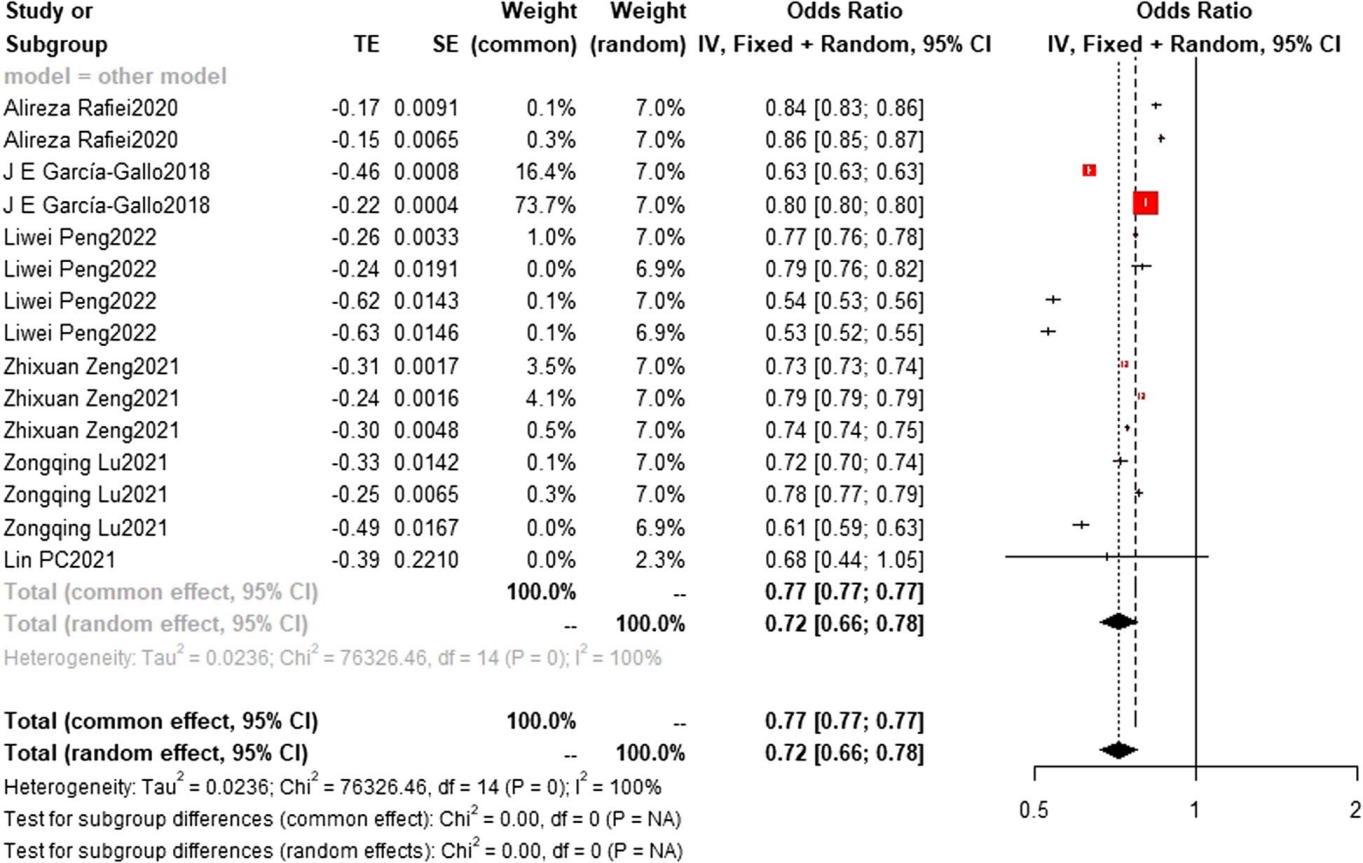
Train set c-index. (Other models include GRU, LSTM, SIRS, SIC, SGB, OASIS, Nomogram, LODS, LDA, CART, MIG, LLI, ET, CS) In train set, the c-index of other models(*n* = 6) is 0.72 (0.66,0.78)

**Fig. 12:**
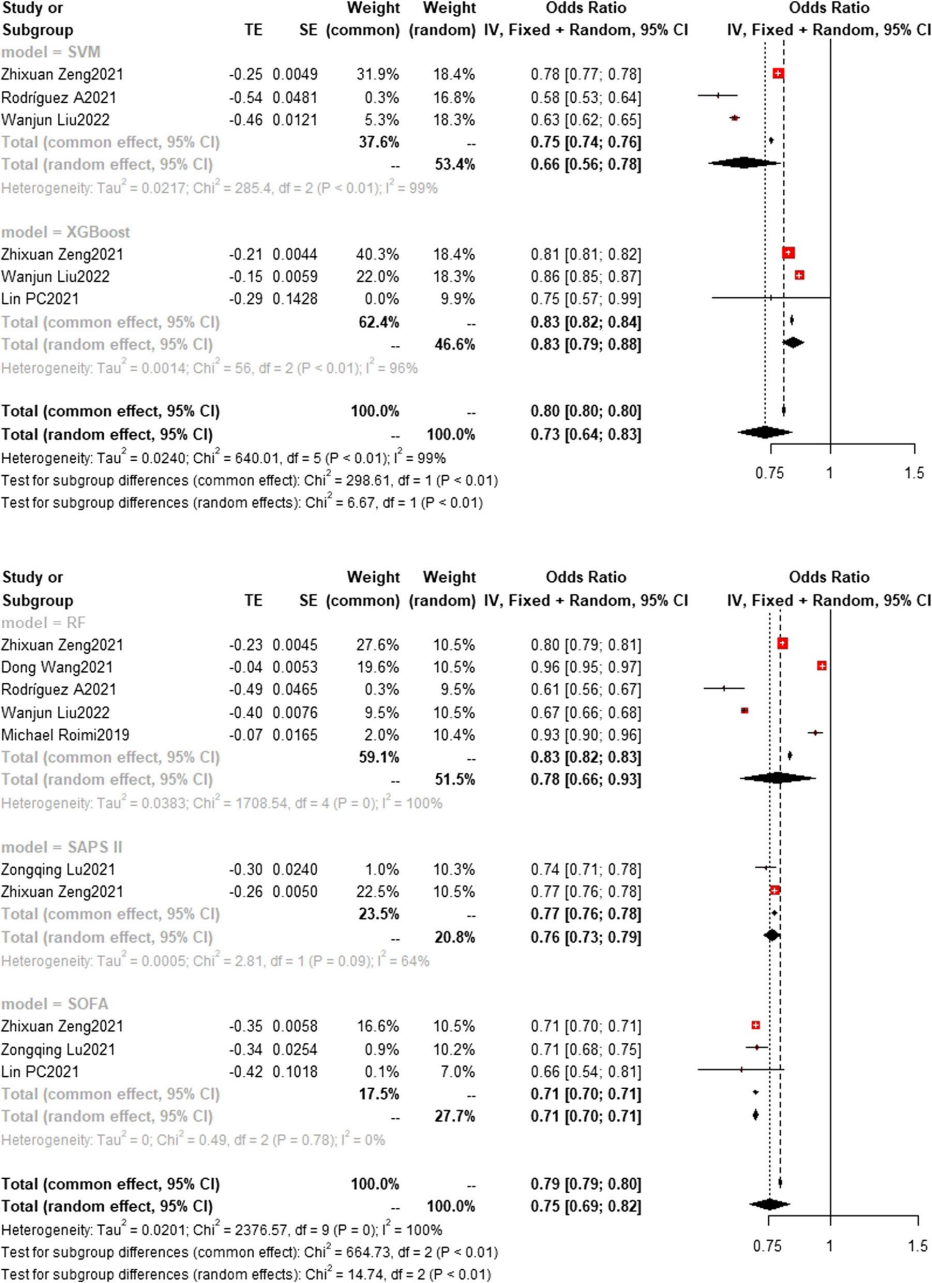
Test set c-index. In the test set, the random forest model was the most frequently employed machine learning model with a c-index of 0.83 (0.82,0.83) in 5 studies. In terms of performance, both the random forest model (*n* = 5, c-index = 0.83 (0.82,0.83)) and XGBoost (*n* = 3, c-index = 0.83 (0.82,0.84)) exhibited similar performance. The rest are SVM(*n* = 3) with c-index 0.66 (0.56, 0.78) SAPS II (*n* = 2) with c-index 0.76(0.73,0.79) and SOFA(*n* = 3) with c-index 0.71(0.70,0.71)

**Fig. 13:**
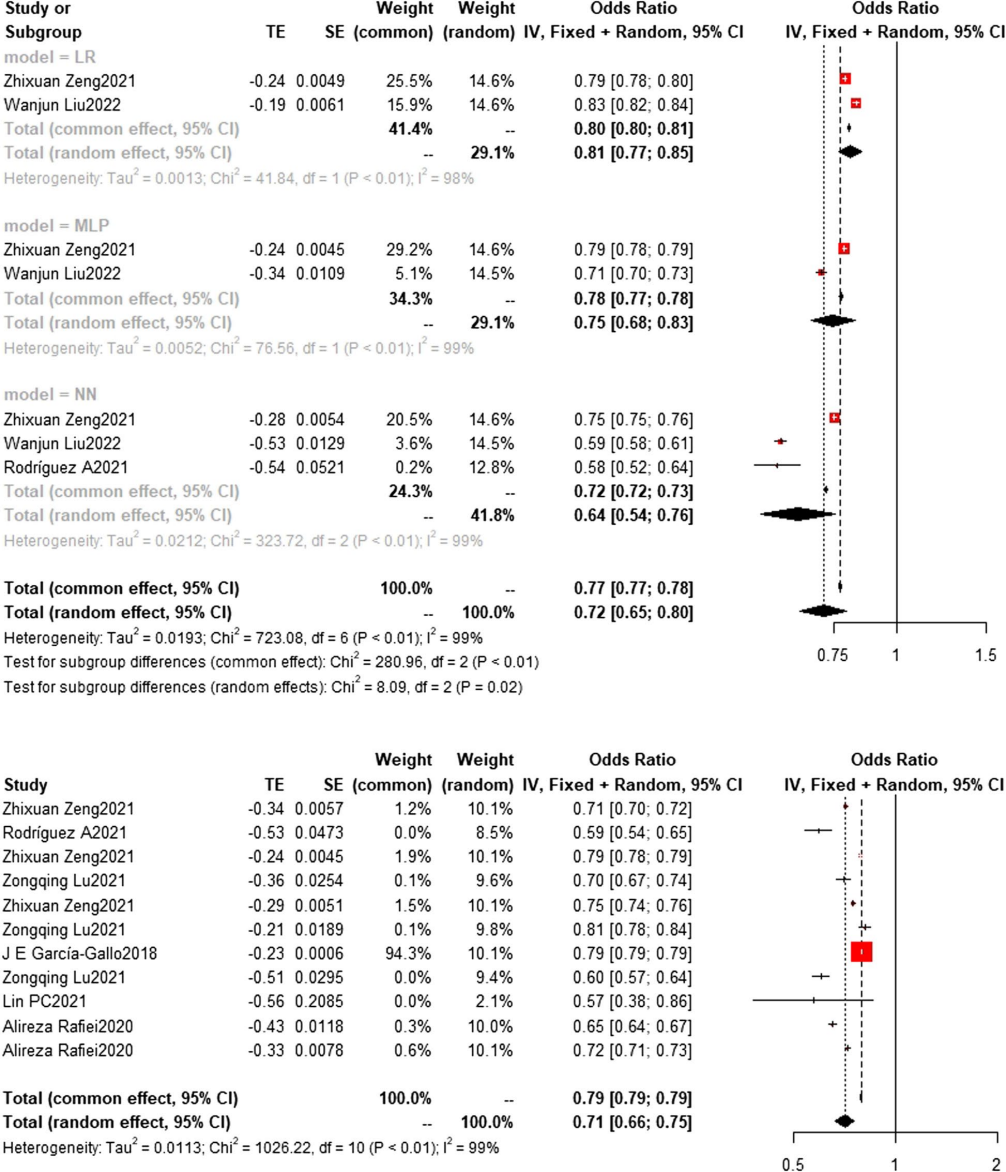
Test set c-index. The LR(*n* = 2), MLP(*n* = 2) and NN(*n* = 3) models showed c-index 0.81(0.77,0.85) 0.75(0.68,0.83) and 0.64(0.54,0.76)

Regarding the c-index results, in the training set, the XGBoost model was the most frequently utilized machine learning model, with a c-index of 0.83 (0.83, 0.84) in 7 studies. The InSight model exhibited the best performance, with a c-index of 0.91 (0.90, 0.93) in 2 studies. On the other hand, in the test set, the random forest model was the most frequently employed machine learning model, with a c-index of 0.83 (0.82,0.83) in 5 studies. In terms of performance, the random forest model (*n* = 5, c-index = 0.83 (0.82,0.83)) and XGBoost model (*n* = 3, c-index = 0.83 (0.82,0.84)) exhibited similar performance. Detailed datasets can be found in Figs. 9, 10, 11, 12 and 13, and the overall results are presented in Supplementary file 3.

## Discussion

The present study investigated 68 prognostic prediction models across 23 studies to assess the potential of machine learning models for predicting sepsis in the ICU. However, the risk of bias assessment revealed a high risk of bias in the analysis domain, which may be attributed to the small sample size, the processing of missing data, and the interpretation of complex data. Therefore, the research findings may have some deviation due to the insufficient sample size.

Sepsis is a severe medical condition that can cause widespread inflammation and damage to vital organs. Early detection and treatment of sepsis are critical for improving patient outcomes and reducing health care costs. ML models can analyse large amounts of patient data, including vital signs, laboratory results, and electronic health records, to detect early signs of sepsis. ML algorithms can provide physicians with real-time recommendations for patient treatment and management based on the latest medical knowledge and patient data. The use of ML models for predicting the onset of sepsis in the ICU has the potential to revolutionize the way in which sepsis is detected, treated, and managed, leading to better patient outcomes and reduced health care costs.

Several studies have explored the potential of machine learning algorithms for predicting sepsis. Heather M et al. [28] developed a machine learning algorithm to predict severe sepsis and septic shock, which can predict, with high specificity, the impending occurrence of severe sepsis and septic shock. Lucas M Fleuren et al. designed a meta-analysis that found that individual machine learning models can accurately predict sepsis onset early, similar to the present study. Nianzong Hou et al. [29] developed an XGBoost model to predict 30-day mortality, which can assist clinicians in tailoring precise management and therapy for patients with sepsis. Dong Wang et al. [13] developed an artificial intelligence algorithm to predict sepsis early, which has shown good predictive ability in Chinese sepsis patients. However, external validation studies are necessary to confirm the universality of this method for the population and in treatment practice.

In this study, we concluded that two machine learning algorithms, the XGBoost and random forest, showed significant advantages in predicting sepsis incidence in ICU patients with higher ACC and c-index values compared to other models in this study, specifically the random forest (test set *n* = 9, acc = 0.911) and extreme gradient boost (test set *n* = 7, acc = 0.957) models. Compared to other studies, this study compared all previous machine learning models for predicting sepsis incidence in ICU patients, including 4,314,145 patients and 26 different machine learning models. This was a large, comprehensive study that strictly followed the PRISMA requirements for systematic evaluation and was methodologically rigorous and scientific. Based on this, we believe that our study is more accurate than previous studies.

The XGBoost and random forest are two machine learning algorithms that showed significant advantages compared to other models in the present study. XGBoost is a popular open-source software library for machine learning that is optimized for speed and scalability, making it one of the most efficient gradient boosting algorithms available. It can handle missing data and noisy data, making it a robust solution for real-world data problems. Random forest is a widely used ensemble machine learning algorithm that combines multiple trees to form a forest and produces a final prediction by aggregating the results from all the trees. These algorithms have been applied in various industries, including finance, health care, and marketing, and have won several machine learning competitions [30]. In our research, the random forest and XGBoost models showed significant advantages compared to other models. We also found other studies using machine learning to predict the incidence of sepsis. Bloch et al. [31] conducted a study using machine learning to predict the onset of sepsis. They found that the support vector machine (SVM) model had the best performance in predicting the onset of sepsis. Compared with this study, the study conducted by Bloch et al. focused on the data of a single medical centre and did not evaluate the data of other medical centres; therefore, the results can only reflect the situation of their single centre, lacking reference value for other regions.

## Conclusion

Machine learning has proven to be an effective tool for predicting sepsis at an early stage. However, to obtain more accurate results, additional machine learning methods are needed. In our research, we discovered that XGBoost and random forest models are the most commonly used models for predicting sepsis incidence in ICU patients, and they exhibit significant performance and accuracy compared to other models. The use of predictive models for early risk assessment has relatively ideal effects in preventing sepsis incidence in ICU patients; however, it still needs further improvement. Therefore, we look forward to more validated machine learning methods based on convenient, noninvasive, or minimally invasive predictive indicators, which may have significant performance and accuracy in predicting sepsis incidence in ICU patients.

## Limitations

This study also has some limitations. First, this study focused on the accuracy of machine learning models and did not include risk factors that lead to the high incidence rate of sepsis in ICU patients. Second, some included models contained special variables related to the diagnosis of sepsis (such as infection indicators), which are valuable for further validation and research in subsequent studies.

### Supplementary Information

**Additional file 1.** PRISMA 2020 Checklist.

**Additional file 2.** 

**Additional file 3.** 

## Data Availability

The datasets used and/or analyzed during the current study are available from the corresponding author on reasonable request.
