# Supplementary material for: Predicting sepsis onset in ICU using machine learning models: a systematic review and meta-analysis
Source: BMC Infect Dis. 2023 Sep 27;23:635. doi: 10.1186/s12879-023-08614-0 (PMC10523763; doi:10.1186/s12879-023-08614-0)
Supplement: Supplementary file 2 — Additional file 2. [file 12879_2023_8614_MOESM2_ESM.docx]

| Database | | Retrieval Formular | | | | | Result | Retrieval Time |
| --- | --- | --- | --- | --- | --- | --- | --- | --- |
| Chchrane Library | | #1 | | (Bloodstream Infection):ti,ab,kw OR (Bloodstream Infections):ti,ab,kw OR (Infection, Bloodstream):ti,ab,kw OR (Pyemia):ti,ab,kw OR (Pyemias):ti,ab,kw OR (Pyohemia):ti,ab,kw OR (Pyohemias):ti,ab,kw OR (Pyaemia):ti,ab,kw OR (Pyaemias):ti,ab,kw OR (Septicemia):ti,ab,kw OR (Septicemias):ti,ab,kw OR (Blood Poisoning):ti,ab,kw OR (Blood Poisonings):ti,ab,kw OR (Poisonings, Blood):ti,ab,kw OR (Poisoning, Blood):ti,ab,kw OR (Severe Sepsis):ti,ab,kw OR (Sepsis, Severe):ti,ab,kw | | | 6117 | 14/11/2022 |
|  |  | #2 | | (sepsis):ti (Word variations have been searched) | | | 3051 |  |
|  |  | #3 | | #1 or #2 | | | 8004 |  |
|  |  | #4 | | (machine learning):ti | | | 771 |  |
|  |  | #5 | | (Learning, Machine):ti,ab,kw OR (Transfer Learning):ti,ab,kw OR (Learning, Transfer):ti,ab,kw | | | 3501 |  |
|  |  | #6 | | #4 or #5 | | | 3501 |  |
|  |  | #7 | | (intensive care unit):ti | | | 2506 |  |
|  |  | #8 | | (Intensive Care Unit):ti,ab,kw OR (Unit, Intensive Care):ti,ab,kw OR (ICU Intensive Care Units):ti,ab,kw | | | 20738 |  |
|  |  | #9 | | #7 or #8 | | | 20738 |  |
|  |  | #10 | | #3 and #6 and #9 | | | 12 |  |
| Database | | Retrieval Formular | | | Result | | | Retrieval Time |
| Embase | | #1 | | 'intensive care unit'/de | 207246 | | | 14/11/2022 |
|  |  | #2 | | 'abdominal sepsis':ab,ti OR 'focal sepsis':ab,ti OR 'intraabdominal sepsis':ab,ti OR 'sepsis syndrome':ab,ti OR 'septic disease':ab,ti OR 'intensive care unit':ab,ti OR 'close attention unit':ab,ti OR 'combined medical and surgical icu':ab,ti OR 'combined surgical and medical icu':ab,ti OR 'critical care unit':ab,ti OR 'general icu':ab,ti OR 'gicu':ab,ti OR 'gicus':ab,ti OR 'icus intensive care department':ab,ti OR 'intensive care units':ab,ti OR 'intensive therapy unit':ab,ti OR 'intensive treatment unit':ab,ti OR 'medical-surgery icu':ab,ti OR 'medical/surgical icu':ab,ti OR 'medical/surgical icus':ab,ti OR 'medico-surgical icu':ab,ti OR 'mixed medical and surgical icu':ab,ti OR 'mixed surgical and medical icu':ab,ti OR 'respiratory care unit':ab,ti OR 'respiratory care units':ab,ti OR 'special care unit':ab,ti OR 'surgery/medical icu':ab,ti OR 'surgical-medical icus':ab,ti OR 'surgical/medical icu':ab,ti OR 'unit intensive care':ab,ti | 217196 | | |  |
|  |  | #3 | | #1 or #2 | 298720 | | |  |
|  |  | #4 | | 'sepsis'/de | 191142 | | |  |
|  |  | #5 | | 'bloodstream infection':ab,ti OR 'bloodstream infections':ab,ti OR 'infection, bloodstream':ab,ti OR 'pyemia':ab,ti OR 'pyemias':ab,ti OR 'pyohemia':ab,ti OR 'pyohemias':ab,ti OR 'pyaemia':ab,ti OR 'pyaemias':ab,ti OR 'septicemia':ab,ti OR 'septicemias':ab,ti OR 'blood poisoning':ab,ti OR 'blood poisonings':ab,ti OR 'poisonings, blood':ab,ti OR 'poisoning, blood':ab,ti OR 'severe sepsis':ab,ti OR 'sepsis, severe':ab,ti | 50965 | | |  |
|  |  | #6 | | #4 or #5 | 223967 | | |  |
|  |  | #7 | | 'machine learning'/de | 73128 | | |  |
|  |  | #8 | | 'learning, machine':ab,ti OR 'transfer learning':ab,ti OR 'learning, transfer':ab,ti | 5868 | | |  |
|  |  | #9 | | #7 or #8 | 77044 | | |  |
|  |  | #10 | | #3 and #6 and #9 | 150 | | |  |
| Database | Retrieval Formular | | | | | Result | | Retrieval Time |
| PubMed | #1 | | "Machine Learning"[MeSH Terms] | | | 50896 | | 14/11/2022 |
|  | #2 | | "learning machine"[Title/Abstract] OR "transfer learning"[Title/Abstract] OR "learning transfer"[Title/Abstract] | | | 5510 | |  |
|  | #3 | | #1 or #2 | | | 54419 | |  |
|  | #4 | | "Sepsis"[MeSH Terms] | | | 138158 | |  |
|  | #5 | | "bloodstream infection"[Title/Abstract] OR "bloodstream infections"[Title/Abstract] OR "infection bloodstream"[Title/Abstract] OR "Pyemia"[Title/Abstract] OR "Pyemias"[Title/Abstract] OR "Pyohemia"[Title/Abstract] OR "Pyohemias"[Title/Abstract] OR "Pyaemia"[Title/Abstract] OR "Septicemia"[Title/Abstract] OR "Septicemias"[Title/Abstract] OR "blood poisoning"[Title/Abstract] OR "blood poisonings"[Title/Abstract] OR (("poisoned"[All Fields] OR "Poisoning"[MeSH Terms] OR "Poisoning"[All Fields] OR "Poisonings"[All Fields] OR "Poisoning"[MeSH Subheading] OR "poisonous"[All Fields] OR "poisons"[Pharmacological Action] OR "poisons"[MeSH Terms] OR "poisons"[All Fields] OR "poison"[All Fields]) AND "Blood"[Title/Abstract]) OR "poisoning blood"[Title/Abstract] OR "severe sepsis"[Title/Abstract] OR "sepsis severe"[Title/Abstract] | | | 66737 | |  |
|  | #6 | | #4 or #5 | | | 183359 | |  |
|  | #7 | | "Intensive Care Units"[MeSH Terms] | | | 102312 | |  |
|  | #8 | | "intensive care unit"[Title/Abstract] OR "unit intensive care"[Title/Abstract] OR "icu intensive care units"[Title/Abstract] | | | 124113 | |  |
|  | #9 | | #7 or #8 | | | 177935 | |  |
|  | #10 | | #3 and #6 and #9 | | | 74 | |  |
| Database | | Retrieval Formular | | | Result | | | Retrieval Time |
| Web of science | | #1 | | TI=(Machine learning) | 84979 | | | 14/11/2022 |
|  |  | #2 | | (TS=(Learning, Machine)) OR (TS=(Transfer Learning)) OR (TS=(Learning, Transfer)) | 758603 | | |  |
|  |  | #3 | | #1 or #2 | 758603 | | |  |
|  |  | #4 | | TI=(sepsis) | 58617 | | |  |
|  |  | #5 | | (TS=(Bloodstream Infection)) OR (TS=(Bloodstream Infections)) OR (TS=(Infection, Bloodstream)) OR (TS=(Pyemia)) OR (TS=(Pyemias)) OR (TS=(Pyohemia)) OR (TS=(Pyohemias)) OR (TS=(Pyaemia)) OR (TS=(Pyaemias)) OR (TS=(Septicemia)) OR (TS=(Septicemias)) OR (TS=(Blood Poisoning)) OR (TS=(Blood Poisonings)) OR (TS=(Poisonings, Blood)) OR (TS=(Poisoning, Blood)) OR (TS=(Severe Sepsis)) OR (TS=(Sepsis, Severe)) | 227452 | | |  |
|  |  | #6 | | #4 or #5 | 217800 | | |  |
|  |  | #7 | | TI=(Intensive care unit) | 49855 | | |  |
|  |  | #8 | | (TS=(Intensive Care Unit)) OR (TS=(Unit, Intensive Care)) OR (TS=(ICU Intensive Care Units)) | 245689 | | |  |
|  |  | #9 | | #7 or #8 | 245689 | | |  |
|  |  | #10 | | #3 and #6 and #9 | 186 | | |  |
