# Supplementary material for: Predicting sepsis onset in ICU using machine learning models: a systematic review and meta-analysis
Source: BMC Infect Dis. 2023 Sep 27;23:635. doi: 10.1186/s12879-023-08614-0 (PMC10523763; doi:10.1186/s12879-023-08614-0)
Supplement: Supplementary file 3 — Additional file 3. [file 12879_2023_8614_MOESM3_ESM.docx]

| Outcome | Model | Training | | | | | Test | | | | |
| --- | --- | --- | --- | --- | --- | --- | --- | --- | --- | --- | --- |
|  |  | n | Total | ACC | low | up | n | Total | ACC | low | up |
| accuracy | DL | 3 | 139348 | 0.998 | 0.095 | 1.000 | 3 | 399592 | 0.830 | 0.814 | 0.845 |
|  | GBT | 2 | 440064 | 0.740 | 0.386 | 0.928 | 4 | 629219 | 0.770 | 0.597 | 0.884 |
|  | InSight | 2 | 41940 | 0.853 | 0.515 | 0.969 | —— | —— | —— | —— | —— |
|  | LR | 6 | 258568 | 0.796 | 0.718 | 0.857 | 4 | 629219 | 0.770 | 0.597 | 0.884 |
|  | MEWS | 3 | 113191 | 0.670 | 0.565 | 0.760 | —— | —— | —— | —— | —— |
|  | MLP | 3 | 102419 | 0.774 | 0.621 | 0.877 | —— | —— | —— | —— | —— |
|  | NB | 2 | 116906 | 0.792 | 0.718 | 0.851 | —— | —— | —— | —— | —— |
|  | NN | 4 | 126866 | 0.769 | 0.571 | 0.893 | 4 | 85576 | 0.712 | 0.491 | 0.864 |
|  | RF | 9 | 579839 | 0.911 | 0.485 | 0.991 | 7 | 492278 | 0.795 | 0.638 | 0.895 |
|  | SOFA | 6 | 184607 | 0.588 | 0.460 | 0.706 | 3 | 264135 | 0.784 | 0.737 | 0.825 |
|  | SVM | 4 | 132760 | 0.788 | 0.635 | 0.889 | 3 | 51104 | 0.804 | 0.687 | 0.885 |
|  | XGBoost | 6 | 149134 | 0.970 | 0.487 | 0.999 | 4 | 56122 | 0.727 | 0.489 | 0.881 |
|  | Other model | 16 | 1911911 | 0.649 | 0.540 | 0.745 | 14 | 1956569 | 0.715 | 0.614 | 0.798 |
|  | DL | 2 | 62808 | 0.74 | 0.52 | 1.05 | —— | —— | —— | —— | —— |
|  | InSight | 2 | 80580 | 0.81 | 0.63 | 1.05 | —— | —— | —— | —— | —— |
| C-index | LR | 4 | 176670 | 0.81 | 0.75 | 0.86 | 2 | 95322 | 0.81 | 0.77 | 0.85 |
|  | RF | 6 | 478481 | 0.74 | 0.66 | 0.84 | 5 | 124768 | 0.78 | 0.66 | 0.93 |
|  | SAPS II | 3 | 114929 | 0.75 | 0.70 | 0.80 | 2 | 64812 | 0.76 | 0.73 | 0.79 |
|  | SOFA | 4 | 78293 | 0.62 | 0.56 | 0.69 | 3 | 73108 | 0.71 | 0.70 | 0.71 |
|  | MLP | 3 | 72506 | 0.79 | 0.65 | 0.97 | 2 | 95322 | 0.75 | 0.68 | 0.83 |
|  | NN | 4 | 136362 | 0.68 | 0.59 | 0.79 | 3 | 100344 | 0.64 | 0.54 | 0.76 |
|  | SVM | 3 | 77386 | 0.67 | 0.57 | 0.78 | 3 | 100344 | 0.66 | 0.56 | 0.78 |
|  | XGBoost | 7 | 568832 | 0.83 | 0.83 | 0.84 | 3 | 103618 | 0.83 | 0.79 | 0.88 |
|  | Other model | 15 | 1908926 | 0.77 | 0.77 | 0.77 | 11 | 263008 | 0.79 | 0.79 | 0.79 |

Other model=GRU(gated recurrent neural unit)、LSTM(Long Short Term Memory)、SIRS (systemic inflammatory response syndrome score)、SIC (sepsis-induced coagulopathy score)、SGB(stochastic gradient boosting)、OASIS (oxford acute severity of illness score)、Nomogram、LODS(logistic organ dysfunction score)、LDA(latent dirichlet allocation)、CART(classification and regression tree) MIG, LLI, ET, CS

DL;DT(Deep learning; Decision Tree), GBT(Gradient Boost Tree), LR(Logistic regression), MEWS(MEWS, Modified Early Warning Score) MLP (Multilayer Perceptron) NB(Naïve Bayes) NN (Neuro network) SVM(Supported Vector Machine) XGBoost (eXtreme gradient boosting)、NAVOY
